# How does the mitotic index impact patients with T1 melanoma? Comparison between the 7th and 8th edition of the American Joint Committee on Cancer melanoma staging system^⋆^^⋆⋆^

**DOI:** 10.1016/j.abd.2020.03.020

**Published:** 2020-09-18

**Authors:** Amanda Zorzetto Antonialli, Eduardo Bertolli, Mariana Petaccia de Macedo, Clovis Antonio Lopes Pinto, Vinicius Fernando Calsavara, João Pedreira Duprat Neto

**Affiliations:** a AC Camargo Cancer Center, São Paulo, SP, Brazil; b Hospital Sírio Libanês, São Paulo, SP, Brazil

**Keywords:** Melanoma, Mitosis, Neoplasm staging, Sentinel lymph node biopsy

## Abstract

**Background:**

The mitotic index is no longer used to classify T1 melanoma patients into T1a and T1b, so it should not be used to indicate sentinel node biopsy in these patients.

**Objectives:**

To evaluate patients with T1 melanoma who underwent sentinel lymph node biopsy and to compare those who were classified as T1a with those classified T1b, according to the 7th and 8th Edition of the melanoma staging system, regarding a positive biopsy result. The authors also aimed to assess whether there is any difference in the results in both staging systems.

**Material and methods:**

This was a retrospective analysis of 1213 patients who underwent sentinel lymph node biopsy for melanoma, from 2000 to 2015, in a single institution.

**Results:**

Of 399 patients with thin melanomas, 27 (6.7%) presented positive sentinel lymph nodes; there was no difference in positivity for sentinel node biopsy when comparing T1a *vs*. T1b in both staging systems. Furthermore, the clinical results were also similar between the two groups. However, in the complete cohort analysis, the mitotic index was associated with positivity for sentinel lymph node biopsy (p < 0.0001), positivity for non-sentinel lymph node (p < 0.0001), recurrence-free survival (p < 0.0001), and specific melanoma survival (p = 0.023).

**Study limitation:**

Unicentric study.

**Conclusion:**

The mitotic index was shown to be a very important prognostic factor in the present study, but it was not observed in patients classified as T1. The mitotic index should no longer be used as the only reason to refer sentinel lymph node biopsy in patients with thin melanoma.

## Introduction

Since January 2018, the 8th edition of the melanoma staging system proposed by the American Joint Committee on Cancer (AJCC) has been incorporated into clinical practice.^1^ One of the differences of this new staging system is the classification of T1, which no longer divides patients into T1a and T1b according to the mitotic index, as in the 7th edition.^2^

In the new staging system, T1a corresponds to patients with Breslow thickness less than 0.8 mm without ulceration; while T1b corresponds to patients with Breslow thickness less than 0.8 mm with ulceration and thicknesses between 0.8–1.0 mm, with or without ulceration. With this new classification, the consensus for sentinel lymph node biopsy (SLNB) proposes that there is no indication for T1a patients, while T1b patients should be considered for the procedure, since the probability of a positive SLNB in these cases ranges from 5% to 10%.^3^, ^4^

However, since the mitotic index was used as a criterion to propose SLNB in patients with thin melanomas in the 7th edition, and still remains as a prognostic factor, should surgeons continue to use this criterion to indicate sentinel lymph node surgery after the introduction of the new staging system?

The primary objective of this study was to evaluate patients with T1 melanoma who underwent SLNB and compare then T1a and T1b groups, as classified by the 7th and 8th editions, regarding sentinel lymph node positivity. It also aimed to assess the positivity of non-sentinel lymph nodes (NSLN) in patients undergoing lymphadenectomy.

The secondary objective was to compare recurrence-free survival (RFS) and melanoma-specific survival (MSS) in patients with T1 melanoma according to both staging systems.

## Material and methods

This is a retrospective analysis of patients who underwent SLNB between 2000 and 2015 at a single institution. The study was approved by the local Research Ethics Committee (Project 2183/16) and this service routine for SLNB has been previously described in the literature.^5^, ^6^

Among patients who had underwent SLNB, only T1 patients were considered, and were divided into T1a and T1b according to the 7th and 8th edition of the AJCC staging system. The chi-squared test and Fisher’s exact test were used to compare the groups regarding SLNB and NSLN positivity, with a significance level of 0.05. Kaplan-Meyer curves were used to assess clinical outcomes in both scenarios, and the non-parametric logrank test was used to assess significance.

The same analyses were also performed on the complete cohort of patients to assess the impact of the mitotic index independently of the T category of the TNM classification for malignant melanoma. Cox regressions were used to assess RFS and MSS.

## Results

Between 2000 and 2015, 1213 patients underwent SLNB at the institution. In this cohort, 411 patients had thin melanomas, but 12 did not present information in the pathology report necessary for staging and were excluded from the analysis; therefore, 399 patients with thin melanomas undergoing SLNB were assessed. In 27 cases (6.7%), SLNB was positive; none of them presented NSLN in lymphadenectomy, which was the conduct of choice during the study period.

**Fig. 1:**
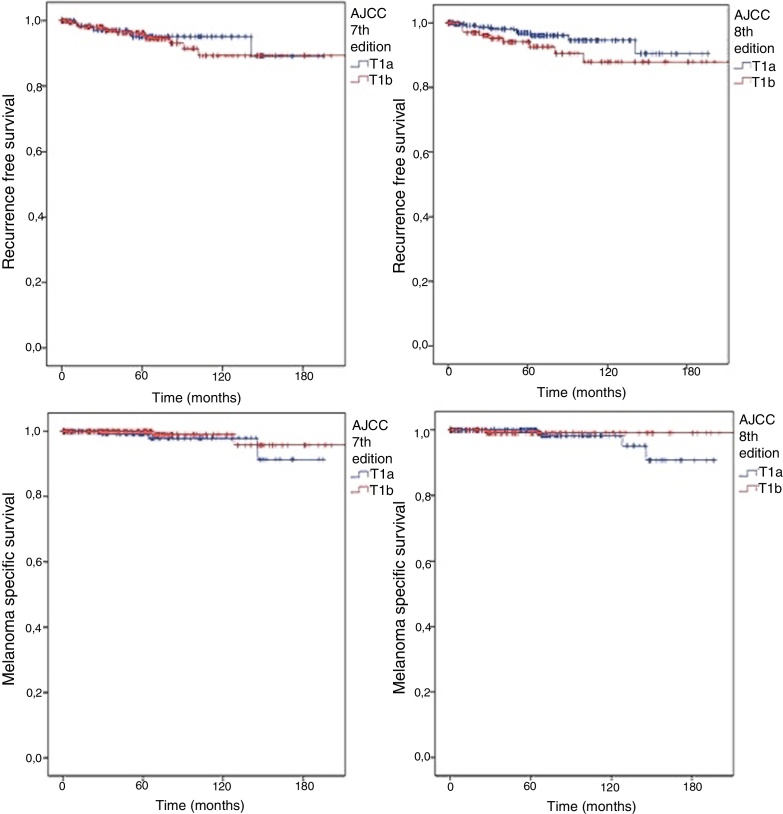
Kaplan Meyer curves for RFS according to (A) 7th edition (logrank = 0.659) and (B) 8th edition (logrank = 0.135); and for MSS according to (C) 7th edition (logrank = 0.363) and (D) 8th edition (logrank = 0.414) for patients with thin melanomas undergoing sentinel lymph node biopsy from 2000 to 2015.

In accordance with the 7th edition, 244 (62.2%) would be classified as T1b for presenting ulceration and/or a mitotic index other than 0, regardless of the scale used (mm^2^ or high power field [HPF]). There was no significant difference in SLNB positivity (OR for T1b: 1.888; 95% CI: 0.779–4.576; p = 0.22). According to the 8th edition classification, the number of T1b patients was lower (14.8%−37.1%) and once again no significant difference was observed between T1a and T1b (OR for T1b: 1.388; 95% CI: 0.631–3.052; p = 0.54). The outcomes were also similar, regardless of the AJCC classification adopted (Fig. 1). In both scenarios, no difference was observed in RFS (logrank = 0.659 for 7th edition and 0.135 for 8th edition) or in MSS (logrank = 0.363 for 7th edition and 0.414 for 8th edition).

However, as classification according to the 8th edition presented different survival curves based on the mitotic index, the authors decided to assess this characteristic in the complete series. Among 1213 patients undergoing SLNB, the mitotic index was statistically different not only in those with positive SLNB (OR = 1.076; 95% CI: 1.054–1.099; p < 0.0001), but also in those with NSLN in lymphadenectomy (OR = 1.067; 95% CI: 1.032–1.103; p < 0.0001).

The mitotic index was also statistically significant in relation with RFS, both in simple (HR = 1.046; 95% CI: 1.038–1.053; p < 0.0001) and in multiple Cox regression (HR = 1.022; 95% CI: 1.010–1.034; p < 0.0001), along with other characteristics of the primary tumor. MSS analysis also demonstrated the significance of the mitotic index in the models of simple (HR = 1.050; 95% CI: 1.040–1.060; p < 0.0001) and multiple regression (HR = 1.020; 95%: CI 1.003–1.038; p = 0.023).

**Fig. 2:**
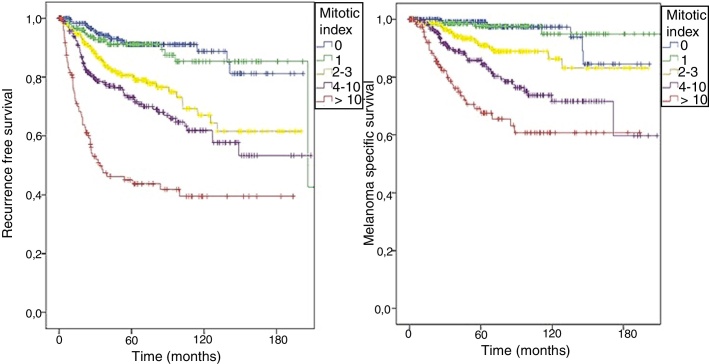
Kaplan Meyer curves for (A) RFS (logrank < 0.0001) and (B) MSS (logrank < 0.0001) according to the mitotic index values in patients with melanoma who underwent sentinel node biopsy from 2000 to 2015.

As shown in the 8th edition of AJCC, the patients in this cohort were also stratified; the RFS (logrank < 0.0001) and MSS (logrank < 0.0001) curves were different according to the value of the mitotic index (Fig. 2).

## Discussion

Updates to staging systems for solid tumors may cause changes in daily clinical practice. In the 8th edition of AJCC staging for melanoma, one of these changes was made in category T1.

The distinction between T1a and T1b is no loger based on mitotic index, as it was in the 7th edition.^2^ This alteration impacts mainly when considering candidates for SLNB, since some patients with thin melanoma have undergone sentinel lymph node surgery based only on the mitotic index.^7^ Now, even though the new consensus no longer uses mitotic index as a criterion for SLNB, some surgeons may question whether they should really change their practice.

The present data showed that, regardless of the staging system adopted, the mitotic index was not associated with SLNB positivity, which suggests that it can be abandoned in this scenario. Moreover, the SLNB positivity in T1 patients was 6.7%, and none of them had a positive NSLN. This reinforces the need to reassess SLNB in T1b patients considering the risks and benefits of the procedure.^3^, ^4^

However, the present results also show the impact of the mitotic index as a prognostic factor unrelated to T staging. It was associated with SLNB and NSLN positivity. In light of the results presented in the De-COG Trial and MSLT-II, much has been discussed about who should receive lymphadenectomy.^8^, ^9^, ^10^ The authors believe that the mitotic index can be used in mathematical tools such as nomograms and algorithms to identify these patients.^11^, ^12^

The main limitation of the present study is probably the low number of patients included, since it was conducted in a single center. However, the present results showed that as the mitotic index increases, this represents a worse prognosis, similar to the survival curves presented in the 8th edition of the AJCC staging system.^1^ The authors believe that this cohort is representative and reinforces the importance of assessing the mitotic index, which should be stated in the pathology reports for melanoma.^13^

## Conclusions

Although the mitotic index is an important prognostic factor in the present analysis, it was not observed in patients with T1 melanoma. As presented in the current consensus, the authors agree that the mitotic index should not be used as the sole criterion to indicate SLNB in patients with thin melanomas. Moreover, the present results suggest that even T1b patients should not receive routine SLNB, since positivity is low and no patient had additional compromised lymph nodes.

## Financial support

None declared.

## Authors’ contributions

Amanda Zorzetto Antonialli: Approval of the final version of the manuscript; conception and planning of the study; elaboration and writing of the manuscript; obtaining, analyzing, and interpreting the data; critical review of the literature; critical review of the manuscript.

Eduardo Bertolli: Statistical analysis; approval of the final version of the manuscript; conception and planning of the study; elaboration and writing of the manuscript; obtaining, analyzing, and interpreting the data; intellectual participation in propaedeutic and/or therapeutic conduct of studied cases; critical review of the literature; critical review of the manuscript.

Mariana Petaccia de Macedo: Approval of the final version of the manuscript; conception and planning of the study; elaboration and writing of the manuscript; obtaining, analyzing, and interpreting the data; intellectual participation in propaedeutic and/or therapeutic conduct of studied cases; critical review of the literature; critical review of the manuscript.

Clovis Antonio Lopes Pinto: Approval of the final version of the manuscript; conception and planning of the study; elaboration and writing of the manuscript; obtaining, analyzing, and interpreting the data; effective participation in research orientation; intellectual participation in propaedeutic and/or therapeutic conduct of studied cases; critical review of the literature; critical review of the manuscript.

Vinicius Fernando Calsavara: Statistical analysis; approval of the final version of the manuscript; conception and planning of the study; elaboration and writing of the manuscript; effective participation in research orientation; critical review of the manuscript.

João Pedreira Duprat Neto: Approval of the final version of the manuscript; conception and planning of the study; elaboration and writing of the manuscript; obtaining, analyzing, and interpreting the data; effective participation in research orientation; intellectual participation in propaedeutic and/or therapeutic conduct of studied cases; critical review of the literature; critical review of the manuscript.

## Conflicts of interest

None declared.
